# How Do Pollen Allergens Sensitize?

**DOI:** 10.3389/fmolb.2022.900533

**Published:** 2022-06-16

**Authors:** Svetlana V. Guryanova, Ekaterina I. Finkina, Daria N. Melnikova, Ivan V. Bogdanov, Barbara Bohle, Tatiana V. Ovchinnikova

**Affiliations:** ^1^ Science-Educational Center, M. M. Shemyakin & Yu. A. Ovchinnikov Institute of Bioorganic Chemistry, The Russian Academy of Sciences, Moscow, Russia; ^2^ Medical Institute, Peoples’ Friendship University of Russia, The Ministry of Science and Higher Education of the Russian Federation, Moscow, Russia; ^3^ Department of Pathophysiology and Allergy Research, Center for Pathophysiology, Infectiology and Immunology, Medical University of Vienna, Vienna, Austria; ^4^ Department of Biotechnology, I.M. Sechenov First Moscow State Medical University (Sechenov University), Moscow, Russia

**Keywords:** allergen, pollen, sensitization, lipids, allergen-specific antibody, ligand-allergen interaction

## Abstract

Plant pollen is one of the main sources of allergens causing allergic diseases such as allergic rhinitis and asthma. Several allergens in plant pollen are panallergens which are also present in other allergen sources. As a result, sensitized individuals may also experience food allergies. The mechanism of sensitization and development of allergic inflammation is a consequence of the interaction of allergens with a large number of molecular factors that often are acting in a complex with other compounds, for example low-molecular-mass ligands, which contribute to the induction a type 2-driven response of immune system. In this review, special attention is paid not only to properties of allergens but also to an important role of their interaction with lipids and other hydrophobic molecules in pollen sensitization. The reactions of epithelial cells lining the nasal and bronchial mucosa and of other immunocompetent cells will also be considered, in particular the mechanisms of the activation of B and T lymphocytes and the formation of allergen-specific antibody responses.

## 1 Introduction

Allergic diseases (AD) are a global public health problem and, according to experts from the World Allergy Organization, the number of patients with allergic diseases increases by 50% every 10 years (Pawankar et al., 2008) and by 2050 about 4 billion people will suffer from allergy (EAACI et al., 2014).

The mechanism of sensitization and development of allergic inflammation is a consequence of the interaction of a large number of molecular factors with allergens that differ in structure and often are acting in a complex with high- and low-molecular compounds, representatives of the microbial community, as well as under the influence of external environment.

Plant pollen is one of the main sources of allergens causing allergic diseases such as allergic rhinitis, asthma, conjunctivitis and dermatitis. Allergic rhinitis caused by pollen has proven to be the most common allergic pathology in most countries. Currently, the prevalence of allergic rhinitis, depending on the region, is 10%–40% and continues to grow all over the world (Brożek et al., 2017). In Europe, a survey of 140 thousand people aged from 20 to 44 years from 22 countries within the framework of the European Community Respiratory Health Survey (ECRHS) established the prevalence of allergic rhinitis from 9.5% to 40.9% of the total adult population, while 16.2%–44.5% had an increased sensitivity to common aeroallergens (Heinrich et al., 2002). In 2000, the number of people in the United States suffering from allergic rhinitis has been from 20 to 40 million and it has almost doubled in 10 years (Skoner, 2001), thus amounting in 2010 to 60 million people (Meltzer et al., 2012), although allergens are very different in various climatic zones. In a large-scale study by the Global Asthma and Allergy European Network (GA2LEN), a significant correlation was demonstrated between development of allergic diseases and sensitization to pollen. The level of clinically significant sensitization to plant pollen was observed in all countries and amounted to 60% of all patients with allergic pathology (Burbach et al., 2009). Pollen sensitization very often precedes food allergy (Eriksson, 1978; Calkhoven et al., 1987; Bircher et al., 1994), which can be severe (Chivato et al., 1996; Wuthrich and Ballmer-Weber, 2001).

Sensitization to pollen allergens is the most common cause of allergic rhinitis (Asher et al., 2006; WAO, 2016; Brożek et al., 2017; Wang et al., 2018). According to the data of European Academy of Allergy and Clinical Immunology (EAACI) about 21%–32% of population suffer from pollen allergens (Brożek et al., 2017). The report of the International Study of Asthma and Allergies in Childhood (ISAAC) declared the prevalence of plant pollen allergy in children aged from 13 to 14 years old, and in total 22.1% of children have pollen allergy (WAO, 2016).

Sensitization to pollen allergens that occurs in early childhood may remain unnoticed and afterwards may cause allergic diseases (Wong et al., 2012; Lourenço et al., 2020). When analyzing the results of 6,220 skin prick tests, European doctors also found an increase in sensitization to pollen allergens with age. Over the past 20 years, the total amount of sensitization to weed pollen has increased significantly, as well as an increase in polysensitization, mainly in young patients (Forkel et al., 2020).

Allergenic pollen comes from three main plant groups—trees, grasses, and weeds. Allergy patients may be sensitive to pollen from one or more plant taxa. Panallergens presenting in plant pollen sensitize the human immune system and contribute to the development of reactions to several allergens (Bohle, 2007; Westman et al., 2020). In a study of 936 patients with allergic rhinitis, 52% were found to be allergic to tree, mugwort and grass pollen, while monoallergenic sensitization to birch pollen was observed in 24%, to mugwort pollen—in 10%, and to grass pollen—in 4% (Osterballe et al., 2005). As a result of sensitization, there is a predisposition not only to food allergies (Osterballe et al., 2005), but also to asthma (Taylor et al., 2007; Shrestha et al., 2018; De Roos et al., 2020) and dermatitis (Wassmann - Otto et al., 2018; Kim et al., 2019). A differentiated distribution of clinically significant sensitization was demonstrated depending on the type of allergen and country, but it turned out that birch pollen was the most common allergen in Europe; more than 20% of Europeans with allergic pathology suffer from birch pollen allergy (Burbach et al., 2009). The prevailing effect of birch pollen on the course of pollinosis was also found in China: 83% of people suffering from pollinosis in spring were sensitized to birch pollen (Li et al., 2020). In general, over 100 million people worldwide are allergic to the birch pollen possibly due to a high content of pollen grains in the air during pollination (more than 20,000 per 1 m^3^) (Aglas et al., 2018; D'Amato et al., 2007).

It is now known that weather, climate, environmental conditions, and human activities have a significant impact on the amount and diversity of pollen (Radauer and Breiteneder, 2006; Damialis et al., 2019). Climate change affects the course of pollen seasons, the distribution of plant species and the content of allergens in pollen grains. In turn, human activities have led both to the introduction of allergenic nonendemic plants and to increased levels of air pollutants that cause respiratory allergies (Ghiani et al., 2012). Pollutants can break down the pollen cell wall, allowing the allergen to be released into the environment and enter the lower respiratory tract (Sinha et al., 2014). On the other hand, the allergenic potential of allergens can be increased by contact with chemicals.

The present review summarizes contemporary outlooks on pollen sensitization of immune system resulting in allergic diseases.

## 2 Characterization of Pollen Allergens

### 2.1 Classification of Pollen Allergens

Plant allergens from tree, grass and weed pollen belong to diverse protein classes the most significant of which are Bet v 1 homologs, lipid transfer proteins (LTPs), profilins, polcalcins, β-expansins and group 5 allergens. Due to a high structural homology of the same class representatives, as well as owing to the presence not only in tree and grass pollen, but also in plant food, some of the above mentioned allergens are panallergens causing pollen-pollen and pollen-food cross-allergic reactions (Osterballe et al., 2005; Bohle, 2007; Sinha et al., 2014; Vizzardelli et al., 2020; Westman et al., 2020). This happens as a result of the similarity of the epitopes of the allergens of the same class and cross-linking of IgE on granulocytes, which are formed in response to sensitization.

Bet v 1 homologs as well as LTPs belong to a large family of pathogenesis-related proteins (PR-proteins, PR-10 and PR-14, respectively). These proteins are present constitutively in different plant organs and tissues as well as in pollen, but the induction of their synthesis occurs in response to stress. Another common characteristic of Bet v 1 homologs and LTPs is the presence of an intrinsic hydrophobic cavity in their structure which allows these proteins to bind different hydrophobic molecules (Finkina et al., 2017). Ligand-biding capacity and specificity may differ for allergens of the same class conforming to volume and shape of a hydrophobic cavity. Recent data showed that ligand-binding played an important role in sensitization and manifestation of allergenic properties of Bet v 1 homologs and LTPs (Fujimoto et al., 1998; Chruszcz et al., 2021). IgE antibodies to Bet v 1 cross-react with homologues allergens from pollen of such trees as alder (Aln g 1), hazel (Cor a 1), beech (Fag s 1), chestnut (Cas s 1) and others (Grilo et al., 2021) as well as with Bet v 1-like proteins from nuts, fruits and vegetables, particularly, with the peanut Ara h 8 apple Mal d 1 (Vanekkrebitz et al., 1995; Bohle et al., 2003), carrot Dau c 1 (Zulehner et al., 2017), causing oral allergy syndrome (OAS) (Costa et al., 2022) in 70% of birch allergic patients (Högerle et al., 2022). Symptoms of OAS usually appear immediately after plant food consumption and mainly affect oropharyngeal area, but such severe reactions as anaphylaxis also may take place (Högerle et al., 2022). The spatial structures of Bet v 1-like allergens are similar to each other and consist of both β-sheets and α-helixes forming together Y-shaped hydrophobic cavity (van Loon and van Kammen, 1970; Fujimoto et al., 1998; Pasternak et al., 2006). Bet v 1 homologs bind such hydrophobic ligands as cytokinins, flavonoids, and sterols (Radauer et al., 2008; Clemente et al., 2012; Aglas et al., 2020). It was shown that natural ligands of two major pollen allergens—the birch Bet v 1 and hazel Cor a 1, are flavonoids (quercetin-3-O-sophoroside (Q3OS) and quercetin-3-O-(2″-O-β-D-glucopyranosyl)-β-D-galactopyranoside, respectively), (Table 1). Minor pollen allergens (Bet v 2-7, Ole 2-11, Cor a 6, Jun a 3, Jun a 4, Jun o 4, Jun v 3, Syr v 3) also demonstrate pollen-pollen and pollen-food reactivity. As supposed, these pollen allergens may take part in plant reproduction via binding and storage of functionally inert glycosylated flavonoids (Soh et al., 2019).

**TABLE 1:** Major pollen allergens and cross-reactivity.

Proteins of Pollen allergens	Major allergens	Cross-Reactive allergens			
		Pollen	Fruits	Vegetables	Other
Bet v 1-related proteins	Alder (Aln g 1)		Gold kiwi (Act c 8)	Celery (Api g 1)	Hazelnut (Cor a 1)
	Birch (Bet v 1)		Kiwi (Act d 8)	Carrot (Dau c 1)	Soy (Gly m 4)
	Hornbeam (Car b 1)		Kiwi (Act d 11)	Tomato (Sola l 4)	Mung bean (Vig r 1)
	Chestnut (Cas s 1)		Peanut (Ara h 8) Strawberry (Fra a 1)		
	Hazel (Cor a 1)		Apple (Mal d 1) Apricot (Pru ar 1) Sweet cherry (Pru av 1)		
	Beech (Fag s 1)		Peach (Pru p 1)		
	Hophornbeam (Ost c 1)		Pear (Pyr c 1)		
	Oak (Que a 1)		Red raspberry (Rub i 1)		
Ole-e-1 related proteins	Ash (Fra e 1)	Sweet beet (Beta v 1)			
	Privet (Lig v 1)	Pigweed (Che a 1)			
	Lilac (Syr v 1)	Rye grass (Lol p 11)			
		Timothy grass (Phl p 11)			
		English plantain (Pla l 1)			
		Russian thistle (Sal k5)			
Pectate lyases	Japanese cypress (Cha o 1)	Ragweed (Amb a 1)			
	Japanese cedar (Cry j 1)	Mugwort (Art v 6)			
	Cypress (Cup a 1)				
	Common cypress (Cup s 1)				
	Mountain cedar (Jun a 1)				
	Eastern red cedar (Jun v 1)				

In contrast with Bet v 1 homologs, plant LTPs (namely, the proteins of the first subclass, LTP1s) are true food allergens due to their stability to heating, gastroduodenal digestion and food processing for preservation (Volpicella et al., 2015; Finkina et al., 2017; Petersen et al., 2017). LTPs are the major plant allergens of the Rosaceae family for patients not sensitized to birch pollen (Aruanno et al., 2020). LTP-related allergy may be mediated by primary sensitization with a food allergen with or without contribution of pollen allergy or primary allergic sensitization to pollen (Aruanno et al., 2020; Scheurer et al., 2021). The peach major allergen Pru p 3 belonging to the LTP class causes food-food and food-pollen cross-allergic reactions in many cases.

LTPs have predominantly α-helical structure with internal tunnel-like cavity and can bind a wide range of hydrophobic ligands, including saturated and unsaturated fatty acids (FAs)—decanoic [C10], lauric [C12], myristic [C14], palmitic [C16], stearic [C18], palmitoleic [C16:1, cis-9], oleic [C18:1, cis-9], ricinoleic [C18:1, cis-9, 12-OH], elaidic [C18:1, trans-9], linoleic [C18:2, cis-9,12], and linoleinic [C18:3 cis-9,12,15] acids (Scheurer and Schülke, 2018); different phospholipids - lysophospholipids LLPC [C12], LMPC [C14], LMPG [C14], LPPC [C16] and LPPG [C16], as well as phosphatidylcholine (PC), DMPG [C14/C14]; phytosphingosine [C18], ergosterol, jasmonic acid and others (Charvolin et al., 1999; Volpicella et al., 2015; Scheurer and Schülke, 2018). As proposed, CPT-PHS might play a role in flower and fruit development and the complex of LTP with this ligand might prevent double pollination and defend against herbivores until the seed had fully matured (Cubells-Baeza et al., 2017). Recently chemically identical natural ligands have been identified also for the wheat allergen Tri a 14 and for three pollen allergenic LTPs—mugwort Art v 3, pellitory Par j 2, and olive Ole e 7 (Gonzalez-Klein et al., 2021).

Profilins and polcalcins (or pollen calcium-binding proteins) are ubiquitous pollen allergens, occurring in almost all plant families (Hauser et al., 2010; Rodríguez del Río et al., 2018). At the same time, profilins are present not only in pollen (for example, the birch Bet v 2, the timothy Phl p 12, the mugwort Art v 4), but also in fruits and seeds of different plants (including the apple Mal d 4, the soy bean Gly m 3, the orange Cit s 2, the melon Cuc m 2) and, as above-mentioned Bet v 1 homologs, are responsible for pollen-associated food allergic reactions (Valenta et al., 1992; van Ree et al., 1992; Fäh et al., 1995; Rihs et al., 1999; Reindl et al., 2002; Radauer and Breiteneder, 2006). However, due to the low stability of profilins, as a rule, mild allergic symptoms occur (Offermann et al., 2016). Despite their ubiquity the prevalence of sensitization to pollen allergens of profilin and polcalcin classes is usually low. For example, in contrast with Bet v 1, recognized by IgE from sera of most of birch allergic patients, the profilin Bet v 2 and the calcium-binding proteins Bet v 3 and Bet v 4 are minor allergens with frequencies of sensitization below 20% (Radauer and Breiteneder, 2006). It is important to note that structurally similar profilins as well as calcium-binding proteins (in particular calmodulins and calmodulin-like proteins) are also found in other eukaryotes including humans. As believed, low allergenic potential of profilins and calcium-binding proteins might also be due to the presence of similar proteins in human cells and suppression of the immune response to these allergens (Radauer and Breiteneder, 2006). Profilins in their structure contain both α-helices and β-sheets and can bind a variety of different ligands including actin, proline-rich peptides, polyphosphoinositides and others. It is known that these proteins take part in generation of cytoskeleton, but also may be involved in many complex molecular processes in plants as well as in signal transduction (Radauer and Breiteneder, 2006; Hauser et al., 2010). Polcalcins have a typical α-helix protein fold and contain two (for instance, the birch Bet v 4, the timothy Phl p 7), three (the birch Bet v 3) or four (the prickly juniper Jun o 4, the olive Ole e 8) calcium-binding EF-hand (helix-loop-helix) motifs in their structure (Hauser et al., 2010). It is important to note that the major IgE epitopes of polcalcins are not located in conserved calcium-binding regions (Ricciardi et al., 2022), but calcium binding to EF-hand motifs leads to conformational changes in protein structures and affects stability and IgE-binding capacity of the allergens of this class (Parody et al., 2013). As proposed, in plants polcalcins might take part in the control of intracellular calcium levels during pollen germination (Hauser et al., 2010).

The major grass pollen allergens are β-expansins or the group 1 allergens as well as the group 5 allergens with ribonuclease activity (Hrabina et al., 2008). Phl p 1 and Phl p 5 from timothy grass are well studied allergens of these classes causing IgE reactivity in most of grass pollen allergic patients (Pablos et al., 2016). β-Expansins are found in a wide variety of Poaceae grasses, but not in other taxonomically unrelated plants. Besides, these proteins (the Bermuda grass Cyn d 1, the rye grass Lol p 1, the maize Zea m 1, and others) are the most relevant pollen allergens in tropical and subtropical areas where temperate trees such as birch and beech are absent and the grasses of Panicoideae and Chloridoideae subfamilies are predominate (Aud-In et al., 2019). Unlike β-expansins, the group 5 allergens are present only in grasses of the Pooideae subfamily. Expansins are glycoproteins divided into four families according to the sequence similarity: α–expansins (EXPA), β–expansins (EXPB), expansin-like A (EXLA), and expansin-like B (EXLB). Both α-expansins and β-expansins possess cell wall loosening activity, but only β-expansins accumulate in grass pollen in significant amounts and are relevant allergens, which, due to a low homology, do not cross-react with representatives of other families. Probable biological function of β–expansins is cell wall loosening during growth of the pollen tube towards the ovary (Sampedro and Cosgrove, 2005). The group 5 allergens cause severe asthma attacks in sensitized patients. High allergenic activity of these allergens may be due to the features of their structure which consists of two similar flexibly-connected IgE-reactive domains (Göbl et al., 2017). The group 5 allergens possess the ribonuclease activity and, as supposed, in plants might play a role in pollen germination (Göbl et al., 2017).

Some clinically relevant pollen allergens also belong to the classes of defensin-like proteins and pectate lyases. Along with Bet v 1 homologs and LTPs, plant defensins constitute another class of PR-proteins (PR-12). Defensins are small peptides with a pronounced antimicrobial activity which are present in various organs and play an important role in plant defense from phytopathogens. Some pollen defensins may act as allergens (Guryanova and Ovchinnikova, 2022). Defensin-like proteins containing an additional C-terminal domain enriched by hydroxylated and O-glycated proline residues are present in plant pollen and possess allergenic properties (Finkina and Ovchinnikova, 2018). The mugwort Art v 1 is the most studied pollen allergen of this class. The ragweed Amb a 4, the feverfew Par h 1 as well as some proteins from different *Artemisia* species are characterized as cross-reactive Art v 1-like pollen allergens (Pablos et al., 2019). Pectate lyase allergens are enzymes which cleave galacturonic acid-containing polysaccharide chains and in pollen possibly take part in tissue remodeling and in pollen tube outgrowth. The clinically relevant pollen allergens of this class are the ragweed Amb a 1, the mugwort Art v 6, the cypress Cup a 1, the Mountain cedar Jun a 1, and the Japanese cedar Cry j 1, some of which cross-react to each other (Pichler et al., 2015).

### 2.2 Structure of Pollen Grains

**FIGURE 1:**
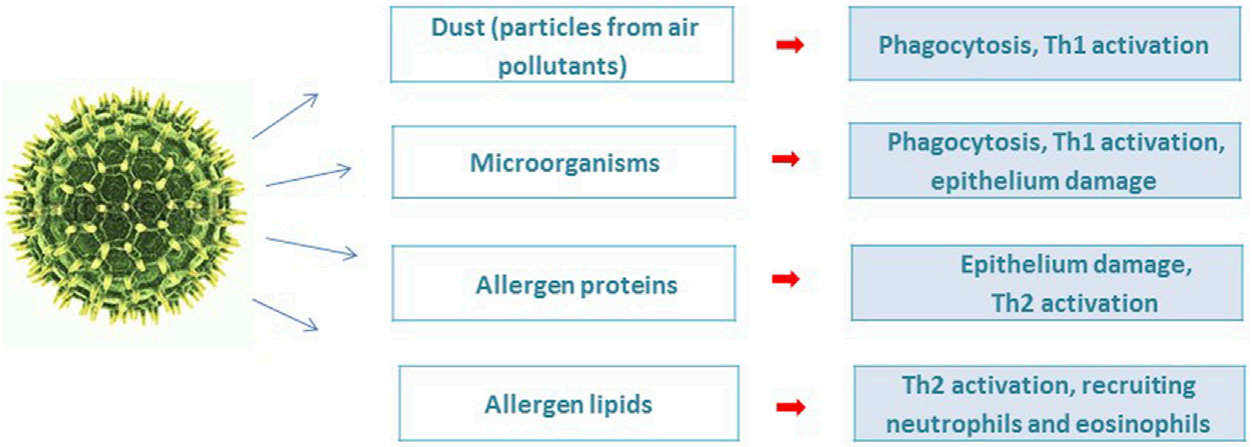
Active compounds associated with pollen.

The pollen grain has a complex architecture in which pollen allergenic proteins are embedded in a heterogeneous matrix of many bioactive molecules that are co-delivered during allergic sensitization. Conventionally, two parts of the pollen grain can be distinguished: the inner, which is represented by proteins, metabolites, lipids, adenosine, flavonoids and the outer, including viruses, bacteria, fungi and particles from air pollutants (Figure 1) (Gilles et al., 2012).

Studies of the transcriptome of pollen, which is a male gametophyte, have shown that the number of expressed genes ranges from 3,400 to 18,500, depending on the plant species (Rutley and Twell, 2015). Comparative analysis revealed that the soybean (Glycine max) pollen transcriptome was enriched with cell wall modifying enzymes, signaling genes and transporters, as well as heat shock proteins, in contrast to Arabidopsis, in which they were not activated (Wang et al., 2008; Haerizadeh et al., 2009). It is noteworthy that after 45 min of cultivation of pollen grains in an *in vitro* medium, the number of activated genes increased threefold (Wang et al., 2008). Thus, during the hydration of pollen that occurs on the mucous membranes when interacting with human biological fluids, the diversity of pollen proteins may increase the pathological process. This assumption can be confirmed by an extremely interesting observation: the occurrence of asthma epidemics, sometimes severe ones, during thunderstorms within the pollen season in different regions. The authors hypothesize the link between thunderstorms and asthma exacerbation, arguing that thunderstorms can concentrate pollen grains at ground level; these grains, after being ruptured by osmotic shock, can release allergenic particles that are inhaled by humans (D'Amato et al., 2017). During the first 20–30 min of a thunderstorm, patients allergic to pollen can inhale a high concentration of allergenic material dispersed in the atmosphere, which in turn causes asthmatic reactions, often severe. Subjects without asthma symptoms but suffering from seasonal rhinitis may also have an asthma attack.

### 2.3 The Role of Ligand-Allergen Interaction in Pollen Sensitization

#### 2.3.1 Involvement of Microbial Ligands in Pollen Sensitization

A metagenomic study of pollen microbiome demonstrates more than a thousand different types of bacteria living on pollen grains of wind-pollinated plants, including birch and grass. Cultivated bacteria, which make up only 5% of all bacteria living on birch pollen grains, have more than 10^6^ cells per gram of pollen (Ambika Manirajan et al., 2016; Alibrandi et al., 2020). Morphological study of pollen grains by scanning electron microscopy showed the presence of biofilms formed by colonies of bacteria and fungi (Colldahl and Nilsson, 1973). Exposure to microorganisms present on pollen can trigger innate immune responses via PRR and modulate the pro-inflammatory response. As a result of the interaction of PAMP of microorganisms with the PRR of immunocompetent cells In addition to protein allergens described above, pollen contains lipids, which also have allergenic properties. induction of cytokines and maintenance of Th1/Th2 balance, and Treg. mechanism may occur (Lin et al., 2020; Tamoutounour et al., 2019; O'Mahony et al., 2008; Kim, 2021; Pascal et al., 2018; Kozlov et al., 2013).

It was shown that homogenate of gram-positive bacteria of *Bacillus* genus found on pollen grains was able to induce maturation of immature dendritic cells (DCs) from grass pollen allergic donors, which led to significantly enhanced expression of costimulatory molecules, such as CD80 and CD86, on surface of DCs (Heydenreich et al., 2012). Thus, stimulation of autologous CD4^+^ T cells by grass pollen allergen-pulsed DCs led to an enhanced production of IL-4, IL-13, IL-10, IL-17, IL-22 and IFN-γ in the presence of the homogenized Gram-positive bacteria compared with T cells stimulated with allergen-pulsed immature DC alone.

#### 2.3.2 Involvement of Lipids and Other Hydrophobic Molecules in Pollen Sensitization

Recently, it was show that not only proteins, but also lipids and other hydrophobic molecules take part in pollen sensitization. The pollen lipid composition of pollen is rich and specific to among plant species, but the main lipid components in most cases are saturated and polyunsaturated fatty acids (FAs), sterol esters, phospholipids, fatty alcohols and sphingolipids (Ischebeck, 2016).

There is an example of immune adaptive recognition of lipids. Some allergic patients were shown to have specific IgE and positive prick cutaneous tests against lipid fraction (phosphatidylcholine (PC) and phosphatidylethanolamine (PE)) isolated from cypress pollen (Behrendt et al., 2001; Traidl-Hoffmann et al., 2002; Plötz et al., 2004; Agea et al., 2005; Traidl-Hoffmann et al., 2005; Gutermuth et al., 2007; Mariani et al., 2007; Gilles et al., 2009a; Bashir et al., 2013; Mittag et al., 2013; Gangwar et al., 2016; Del Moral and Martínez-Naves, 2017), which supported the hypothesis that lipids might be “true” allergens.

**FIGURE 2:**
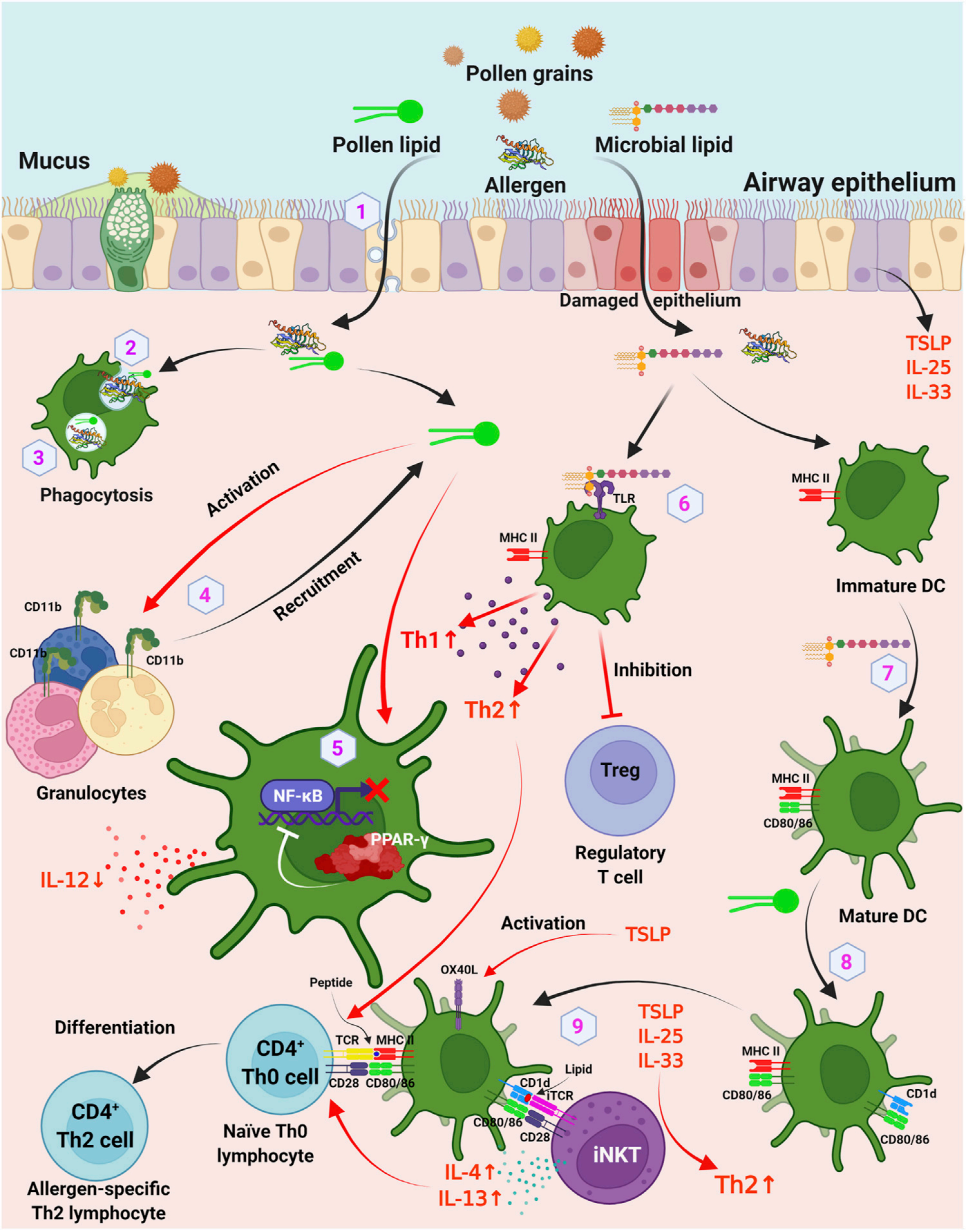
Involvement of lipids and other hydrophobic molecules in pollen sensitization. 1) lipids may influence the transport of pollen allergens through respiratory epithelium by caveolar-dependent way; 2) lipids may affect the uptake rate of the allergen by DCs; 3) lipids may influence the rate of endolysosomal degradation in APCs; 4) pollen lipids act as chemoattractants and activators of granulocytes *via* upregulation of surface integrin CD11b; 5) inhibition of production of IL-12 by DCs *via* PPAR-γ dependent pathways, which leads to inhibition of NF-κB activation and results in reduced IL-12 production; 6) TLR ligands of pollen microbiome enhance Th1 and Th2 responses and decrease induction of Foxp3+ regulatory T cells; 7) microbial lipids induce maturation of immature DCs, which leads to expression of costimulatory CD80 and CD86 on DCs; 8) pollen-derived lipids upregulate CD1d and CD86 molecules on DCs; 9) DCs activate iNKT cells through a CD1d-dependent pathway. DC, dendritic cells; iNKT, invariant natural killer T cells; TLR, toll-like receptor. Figure was created with BioRender.com.

On the one hand, some pollen lipids are bioactive compounds which are capable to cause sensitization, creating prerequisites for Th1/Th2 imbalance towards Th2 way and development of a Th2-mediated allergic response (Figure 2, items 4–9). On another hand, some of them can act as adjuvants that increase or modulate the immune response to the protein allergen which leads to sensitization. Moreover, lipids can increase the allergenic potential of the protein by influencing the rate of its transport through epithelial barriers and processing by immunocompetent cells (Figure 2, items 1–3).

At the same time, it was shown that olive tree pollen lipid extract (polar lipids, diacylglycerols, free fatty acids, and triacylglycerols) upregulated CD1d and CD86 molecules on DCs, which then were able to activate invariant NKT (iNKT) cells through a CD1d-dependent pathway (Abós-Gracia et al., 2013). Today, this mechanism of sensitization by an allergen, involving a cytokine- or CD1d-mediated activation of invariant iNKT (Vα24/Jα18+) -cells by accompanying lipids, is commonly accepted (Scheurer and Schülke, 2018). The mechanism of sensitization, involving CD1d-mediated activation of iNKT cells, was shown for the food allergen Pru p 3 of LTP class (Tordesillas et al., 2017). It is interesting to note that the natural ligand of Pru p 3, the aforementioned 10-OH-camptothecin phytosphingosine (CPT-PHS), was shown to act as an adjuvant and cause allergic sensitization to the allergen. Sensitization of BALB/c mice with the Pru p 3-ligand complex increased the level of Pru p 3-specific IgE antibodies and induced basophil activation as compared to stimulation by Pru p 3 alone which failed to induce sensitization.

Finally, a number of studies have shown that lipids affect the allergenic potential of the proteins. And it is still an open question whether the ability of these proteins to bind lipid molecules is a necessary requirement for this. First, lipids may influence the interactions between pollen allergen and the epithelial barrier of respiratory mucosa. For instance, transport of the lipid-binding allergen Bet v 1 through nasal epithelium of birch pollen allergic patients was proposed to involve an active lipid raft and be mediated by a caveolar-dependent way (Joenväärä et al., 2009). Thus, the primary contact between the allergen and airway epithelium can be mediated by lipids. For example, Bet v 1 has been shown to bind cholesterol and enter the epithelium of allergic patients in cholesterol- and glycolipid-rich caveolae. Second, lipids may influence the uptake of the allergen by antigen presenting cells (APCs). It is known that allergens are processed by DCs *via* endolysosomal compartments, enabling their presentation on MHC II-peptide complexes to T cells. Mustard lipids and phosphatidylglycerol vesicles associate with food allergen Sin a 2 of 11S globulin class was shown to decrease the uptake of the allergen by DCs (Angelina et al., 2016). Third, lipids may also influence the rate of endolysosomal degradation by APCs. Reducing the rate at which allergens are cleaved by cathepsin S has been proposed to skew the immune system towards a Th2 response by preventing premature endosomal degradation and effective MHC II loading. It was demonstrated that pollen-derived ligand of birch Bet v 1 PPE1 can reduce the rate of endolysosomal degradation of the allergen *in vitro* (Soh et al., 2019). However, these data are still to be validated in the experiments *ex vivo* and *in vivo*.

## 3 Sensitization to Pollen Allergens

### 3.1 Epithelial Cells in the Sensitization Process

The first step in the sensitization process of allergic disorders is allergen entry through epithelial surfaces in the nose, gastrointestinal tract or *via* the skin. After inhalation and contact with moist mucosal surfaces pollen are generally believed to release a hydrophilic cocktail consisting of allergens, non-allergenic proteins, and various other bioactive molecules (Gilles et al., 2009b; Bacher et al., 2016; Gilles-Stein et al., 2016; Obersteiner et al., 2016; González Roldán et al., 2019). Early studies have evaluated the time kinetics of allergen release from hydrated pollen grains. Major allergens like Amb a 1 in ragweed or Bet v 1 in birch pollen were found to be liberated within minutes in quantities sufficient for trans-mucosal delivery to sub-epithelial antigen presenting cells (APC) (Marsh et al., 1981; Grote et al., 1993; Deifl et al., 2014). The quantity of allergens transferred through the epithelia depends on the barrier integrity. A disturbed barrier function promotes allergic sensitization forming the “epithelial barrier hypothesis” (Mitamura et al., 2021). Along these lines, different pollen species have been demonstrated to possess enzymatic activity reducing the epithelial integrity (Gunawan et al., 2008; Van Cleemput et al., 2019; Bradbury et al., 2022). Only recently, pollen proteases released from Kentucky Blue Grass, white birch, and hazel pollen were shown to irreversibly disrupt the integrity and anchorage of the columnar respiratory epithelial cell layer, while the basal cell layer resisted their damaging effect (Van Cleemput et al., 2019). However, further investigations are required to elucidate whether the pollen itself and/or pollen-inhabiting microorganisms contribute to such protease activity (McKenna et al., 2017).

As the first line in the defense of invading agents, epithelial cells are equipped with diverse pattern recognition receptors (PRR), such as toll-like receptors (TLR), Nod-like-receptor (NLR), protease-activated receptors (PAR), and scavenger receptors (SR). Upon triggering PRR, epithelial cells synthesize pro-inflammatory cytokines (IL-1, IL-6, IL-8 and TNF-α) and pro-allergic alarmins, e.g., thymic stromal lymphopoietin (TSLP), IL-33, and IL-25 (Bergougnan et al., 2020).

The established crucial role of PRR in the formation of adaptive immune responses has led to the conclusion that allergens which contain specific lipid or carbohydrate ligands directly recruit and activate various PRR pathways on dendritic and stromal cells and thus drive Th2-mediated immune responses (Wills-Karp, 2010). TLR4 has been shown to be necessary and sufficient for the development of Th2 immune responses elicited by several allergens, similar to the mammalian lipid-binding protein MD-2. In this case, the critical factors are exposure time, timing relative to initial sensitization, and cell types (Eisenbarth et al., 2002; Redecke et al., 2004; Tan et al., 2010). In most other allergens containing carbohydrates, strong Th2 immune responses were evoked through the recruitment of C-lectin receptors (MR, DC-SIGN, MR, dectin-2) on dendritic cells (Barrett et al., 2009; Nathan et al., 2009; Hsu et al., 2010). Thus, since complex allergens contain biologically active ligands, the type of subsequent immune response can be determined by the integration of downstream signals initiated by the interaction with PRR. Allergic inflammation develops when there is a lack or loss of anti-inflammatory functions aimed at restricting inflammation through the inactivation of pro-inflammatory mediators (Gushchin, 2020).

Pollen may directly interact with PRR (Scheurer et al., 2015). As one example, pollen from Japanese hop upregulated PAR2 on human airway epithelial cells, which was followed by the production of reactive oxygen species (ROS) and resulted in the synthesis of TSLP (Lee et al., 2014). Similarly, pollen extracts from birch, short ragweed, and timothy grass displayed ROS-elevating activity (Shalaby et al., 2013; Bradbury et al., 2022). TSLP activates dendritic cells (DC) and macrophages to express OX40 ligand (OX40L) or may directly trigger IL-4 and IL-13 production by CD4^+^ T cells. Consequently, this cytokine is regarded as highly relevant for the initiation of Th2-responses. TSLP production may also be induced by mannose residues on allergens, possibly by involving the mannose receptor in allergen recognition and uptake by DC (Al-Ghouleh et al., 2012). The major allergen in *Cupressus arizonica*, Cup a 1, displayed Th2-polarizing activity *via* the induction of IL-33 (Gabriele et al., 2013). This cytokine binds and upregulates ST2, expressed on DC and CD4^+^ T cells, and triggers IL-5 and IL-13 synthesis.

The microbiota of human mucous membranes also contributes to the modulation of the processes of allergic inflammation. It was found that disorder of the microbiome composition led to increased serum IgE concentrations and expanded population of circulating basophils (Hill et al., 2012). Importantly, this link has been shown to be inherent in B cells and dependent on the MYD88 pathway. Moreover, the lung microbiome may also play a role in controlling polarization of the asthma endotype, regulating the balance between Th2 and Th17 patterns. *Enterococcus faecalis* can suppress Th17 immunity and symptoms of allergic airway disease, therefore it is considered a potential therapeutic agent for both asthma and Th17 immunity (Adami and Bracken, 2016). Differences in levels and diversity of the lung microbiome have been divided between healthy individuals and patients with asthma and allergic diseases. In patients with asthma and allergic diseases, the number of proteobacteria was higher; moreover, their presence was associated with the severity of asthma, probably due to the activation of genes associated with Th17 (Hilty et al., 2010; Huang et al., 2011).

Early colonization of the lung mucosa by *Haemophilus influenzae*, *Moraxella catarrhalis*, and *Streptococcus pneumoniae* has been associated with recurrent wheezing and asthma (Bisgaard et al., 2007; Korppi, 2010). By the example of rhinovirus infections of the human nasopharynx, it has been demonstrated that viruses also affect the development of asthma (Teo et al., 2015). An association between the composition of the lung and intestinal microbiome was found and the risk of developing respiratory allergic diseases (Clemente et al., 2012; Wu et al., 2020). This data indicates the involvement of the intestinal mucosa microbiome in the regulation of allergic processes. The debate is still active between two hypotheses: 1) an alteration of microbiota is the result of a disease or 2) an altered microbiome participates in the inception of a disease.

### 3.2 Antigen-Processing and T Cell Epitopes

After trans-epithelial passage allergens are endocytosed and processed by APC, e.g., DC. Both processes are influenced by cytokines and other factors released from epithelial cells following activation by allergens and/or additional pollen compounds. For example, HDM allergens trigger epithelial cells to produce IFN-γ which is known to promote antigen processing and the expression of HLA class II molecules (Vroling et al., 2007).

Also intrinsic characteristics of allergens, such as their 3-dimensional structure, influence their uptake and processing by APC. The effect of structure on endolysosomal fragmentation has been elegantly demonstrated for Bet v 1 by two oppositional approaches. On the one hand, a genetically engineered variant of Bet v 1 with an almost identical amino acid sequence but lacking the typical, compact Bet v 1-fold was more rapidly internalized and processed than Bet v 1 (Kitzmüller et al., 2012). On the other hand, the stabilization of the Bet v 1-structure by ligand-binding in its hydrophobic binding pocket reduced its degradation by lysosomal proteases (Soh et al., 2019). A slow kinetic of endolysosomal fragmentation is characteristic for immunogenic antigens, presumably because it delivers peptides over longer periods which are available for continuous loading of MHC class II molecules (Delamarre et al., 2006). The extent of the peptide-HLA class II complexes on the surface of APC then affects the priming of naïve T cells with lower numbers favoring Th2-polarization (Constant et al., 1995; Freier et al., 2015). Also the type of allergen-derived peptides may contribute to allergenicity as immunodominant peptides which can be loaded to various HLA-phenotypes (promiscuous) are characteristic for major allergens. For instance, Bet v 1 contains one T cell epitope recognized by more than 60% of birch pollen-allergic individuals (Jahn-Schmid et al., 2005). This immunodominant epitope is located in the highly conserved C-terminus of Bet v 1 and thus, similar in various homologous allergens resulting in T cell-cross-reactivity (Ebner et al., 1993; Jahn-Schmid et al., 2005). Other examples of plant pollen-derived allergens are Amb a 1, the major allergen in ragweed, containing three immunodominant epitopes and multiple restriction elements—as well as Art v 1 in mugwort pollen with one dominant T cell epitope restricted to the phenotype HLA-DR01 (Jahn-Schmid et al., 2010; Knapp et al., 2012). Notably, allergens which per se are not considered to initiate sensitization, e.g., the Bet v 1-homologs Mal d 1 (apple) and Api g 1 (celery) lack immunodominant T cell epitopes (Kitzmüller et al., 2015). These results observed for members of the Bet v 1-protein family have been confirmed by very similar results obtained for another family of relevant plant-food allergens, i.e., LTPs (Schulten et al., 2009; Schulten et al., 2011).

It should be noted that in order to capture the allergen, dendritic cells form a network throughout the epithelium of the respiratory tract, including the nose, nasopharynx, large conducting airways, bronchi, bronchioles and alveoli (Humeniuk et al., 2017). It is more likely that nasal dendritic cells are the first to be involved in the processing of allergens, resulting in pollen sensitization. Most pollen grains do not reach the lungs, they are cleared by the mucociliary system. Allergic rhinitis precedes asthma in many patients and is a risk factor for developing asthma. The sensitized epithelium of the respiratory tract contributes to the polarization of macrophages towards M2. Not all phagocytes can present antigens, and their nature determines a way of the response. In addition, the experimental model showed the ability of M2 macrophages and Th2 to produce histamine when interacting with antigens, which explains the mechanism of non-IgE-mediated allergy (Iwasaki et al., 2021).

Only recently, the development of antigen-specific T cell enrichment (ARTE) allowed a more detailed *ex vivo* analysis of allergen-specific effector and regulatory T (Treg) cell responses and suggested that human Th2 and Treg cells react to different proteins in pollen (Bacher et al., 2016). The authors concluded that the dominant immune reaction to inhaled, preferentially particle-associated antigens is tolerance mediated by Treg cells. In contrary, proteins which are rapidly released from inhaled particles, e.g., Bet v 1, fail to actively induce tolerance. Accordingly, the prevalence of Bet v 1-specific Treg cells in non-allergic individuals was reported to be very low. Furthermore, the absence of clonally expanded Bet v 1-specific effector T cells in non-allergic individuals may be interpreted as ignorance of the allergen by the immune system. In support of this hypothesis, recombinant (r) Bet v 1 failed to induce IL-4-producing T cells in IL-4/green fluorescent protein (GFP)-enhanced transcript (4get) mice whereas the aqueous birch pollen extract promoted a Th2-response even when depleted of natural Bet v 1 (Aglas et al., 2018). These findings further underlined that exposure to the entire pollen is relevant for allergic sensitization.

### 3.3 Formation of Immunoglobulins IgE, IgG1

The presence of IgE is the main symptom of an allergic process. Cross-linking of allergen and IgE with the Fcε receptor is necessary for inducing mast cell degranulation. With age, the amount of IgE in people sensitized by pollen of plants is increasing, while the number of allergens that cause a pathological reaction also goes up. Analysis of IgE levels in 123 children in the United States showed that among 2-years-old children with AR 60% had sensitization to weeds, 55% to grasses and 50% to tree pollen, while by the age of 8 years old, 91% were sensitized to weeds, 82% were sensitized to grasses, and 83% were sensitized to tree pollen (Wong et al., 2012).

Study of IgE specificity revealed an association of sensitization to molecular components of pollen allergens with food reactions in patients suffering from atopic dermatitis. In patients suffering from reactions to peanuts, hazelnuts, celery, apple and peach, a significantly higher frequency of sensitization to major pollen allergens Bet v 1, Aln g 1, Phl p 1, 2, 4, 5, 6, 11 was found (Čelakovská et al., 2021a; Čelakovská et al., 2021b). Interestingly, sensitization to Bet v 1 homologues and profilins is associated with mild symptoms (pollen eating syndrome), and sensitization to LTP homologues and seed storage proteins are associated with severe reactions (Čelakovská et al., 2021a).

Immunoglobulin IgE in the serum of healthy people is not formed under the influence of birch pollen. To study the mechanisms of sensitization and regulation of IgE synthesis, the kinetic characteristics of uptake of major and cross-reactive allergens and competitive binding of IgE on the cell surface, were determined. The pathways of internalization of labeled Bet v1 allergens and the cross-reactive celery allergen Api g1 by immature monocyte dendritic cells (iMoDC) from normal donors and patients suffered with birch pollen allergy were studied (Smole et al., 2015). It was found that the internalization of Bet v 1 by iMoDCs from both donor groups had similar kinetics. At the same time, Bet v1 was superior to Api g1 in binding and uptake by the cell surface. The authors of the study proposed a model of receptor-mediated caveolar endocytosis to explain the absorption of allergens by dendritic cells. MoDCs from allergic and healthy donors showed the surface-bound IgE and a pronounced activation of Th2-cytokine and NFκB-dependent genes upon nonspecific cross-linking with the Fcε receptor. In contrast to these IgE-mediated responses, stimulation by Bet v1 increased the levels of Th2 cytokines IL-4 and IL-13, but not NFκB-related genes, in MoDCs of birch pollen allergic donors. Cells from healthy donors either did not respond or showed increased mRNA levels of Th1-mediated chemokines. Moreover, Bet v1 was able to induce the activation of Erk1/2 and p38 MAPK in birch pollen allergy sufferers, but only a minor activation of p38 MAPK was observed in normal donors. Thus, it has been shown that Bet v1 promoted an activation of the Th2 program only in dendritic cells of persons allergic to birch pollen. In addition, in healthy individuals, the predominance of the Th1 response was established as compared to the Th2 response in persons allergic to birch pollen (Smole et al., 2015). The obtained data are consistent with the results of *in vitro* experimental studies on blood mononuclear cells of persons suffering from allergic bronchial asthma. It was found that an activation of the Th1 response using muramyl peptide (an analogue of the bacterial cell wall) shifts the Th1/Th2 balance towards Th1 (Guryanova et al., 2009), explaining the positive effect of muramyl peptide therapy in patients suffering from dermatological and allergic diseases (Kolesnikova et al., 2016; Guryanova et al., 2019). In the experimental model of asthma, it was found that fragments of microorganisms, when used together with an allergen, enhance IgE formation, while the preliminary administration of bacterial innate immunity ligands reduces IgE formation and increases IgG1 and IgG2a (Guryanova et al., 2022).

Thus, one of the strategies for the therapy and prevention of allergic diseases might be the activation of the Th1 response, in which the produced cytokines prevent the formation of Th2, and consequently, IgE formation.

Another strategy for preventing allergic inflammation is the induction of specific IgG antibodies that cross-react with allergens and inhibit binding to IgE due to epitope competition (Subbarayal et al., 2013). It is known that specific immunotherapy with birch pollen (BP-SIT) induces IgG4 antibodies that inhibit IgE binding not only to Bet v1, but also to homologous proteins Mal d1 and Cor a, which cause food allergies. To determine if there is a cross-reactivity of BP-SIT-induced Bet v1-specific IgG4 antibodies with IgE epitopes, IgE and IgG4 levels specific for Bet v1, Mal d1 and Cor a1 were determined in 42 patients allergic to birch pollen before and during BP-SIT. As the result of specific immunotherapy, the concentration of Bet v1-specific IgG4 antibodies, which also reacted with food allergens, significantly increased. At the same time, the level of allergen-specific IgE significantly decreased, sera containing IgG4 antibodies reactive to the food allergen inhibited IgE binding, activation of basophils, and IgE-mediated proliferation of T cells induced by food allergens. The predicted IgE and IgG4 epitopes for all allergens showed a high overlap (Subbarayal et al., 2013).

The therapeutic efficacy of IgG antibodies that block IgE has shown cross-blocking activity against related allergens in *Fagales* pollen. Sublingual immunotherapy with the recombinant Bet v1 during 16 weeks increased cross-reactive serum IgE antibodies and induced IgG1 and IgG4 antibodies with inter- and intraindividual reactivity towards homologues. The cross-blocking bioactivity of these antibodies was highly variable and could not be predicted from protein homology or IgE cross-reactivity. Thus, immunotherapy with the reference allergen Bet v1 induces an individual repertoire of cross-reactive antibodies IgG1 and IgG4 (Grilo et al., 2021).

It should be noted that the level of specific IgE in serum can be maintained for a long time after the cessation of exposure to the allergen (Subbarayal et al., 2013). It was noted that the T-cell response correlated with the annual indices by mugwort pollen. The number of T cells in people allergic to mugwort has dropped sharply since 2004, when there was a sharp decline in annual mugwort pollen indices. Local sensitization to mugwort pollen and serum IgE antibodies specific for Art v1 remained unchanged until 2015, despite the long-term decline in natural exposure to allergens to levels having been too low to stimulate specific T cells (Van Hemelen et al., 2019). Obviously, the existence of memory cells responsible for the synthesis of IgE antibodies is maintained in the body for a long time; their detection and elimination can be an additional therapy strategy.

### 3.4 ILC2-Mediated Sensitization

As new experimental data become available, the above classical concept of the mechanism of development of a Tcells-mediated allergic response turned out to be insufficient to explain the occurrence of an allergic reaction in Rag2^−/−^ mice lacking B- and T-cells (Oboki et al., 2010), or in the absence of IgE (Campana et al., 2008; Chen et al., 2008; Halim et al., 2012).

When studying an influence of cell populations on the appearance and development of allergic reactions in Rag2^−/−^ mice lacking B and T cells, the animals were intranasally injected with allergens isolated from plants and house dust mites and displaying protease activity. Despite the absence of T and B lymphocytes, the animals developed an allergic reaction. In addition, in IL-33 deficient mice, the protease allergen did not induce eosinophilic inflammation after intranasal administration. It turned out that IL-33 activated innate lymphoid cells 2 type (ILC2s), which produce IL-5 and IL-13. IL-5 causes eosinophil degranulation, and IL-13 activates dendritic cells. Thus, a Th2 response occurs in the acute phase of an allergic reaction, and ILC2s are required early in order to mediate the relationship between IL-33 and eosinophilic inflammation. In addition, it was found that mice lacking ILCs (Rag2^−/−^ Il2rg^−/−^) had a marked reduction in the characteristics of allergic pneumonia, eosinophilia and mucus secretion. Experiments have shown an important role of ILC2s in the development of allergic inflammation, and also showed the effect of IL-33 on their activation (Kondo et al., 2008). In this case, the cytokines IL-4, IL-5, IL-9 and IL-13 can be produced not only by Th2, but also by ILC2s, affecting not only the acute phase of the allergic process, but also the chronic one, providing a connection between innate and adaptive immunity (Price et al., 2010; Spits et al., 2013). Thus, the classical Th2-induced immune response was supplemented by new participants—ILC2s.

Innate lymphoid cells (ILCs) have the following characteristics: 1) the absence of antigen-specific receptors, 2) the absence of expression of known markers of immune cell differentiation and 3) lymphoid cell morphology (Spits and Di Santo, 2011). ILCs are divided into five groups depending on their phenotype and functions (Veldhoen et al., 2008). ILC2s produce IL-4, IL-5, IL-9, IL-13 (VivierArtis et al., 2018) and are involved in immune responses caused by parasitic invasions (Cupedo et al., 2009; Pelly et al., 2016) and allergies (Luci et al., 2009), but also serve as systemic regulators of homeostasis (DuPage and Bluestone, 2016; Thome et al., 2016; Lim and Di Santo, 2019). There is a certain degree of plasticity between ILCs, depending on the microenvironment and activating signals, they can change their characteristics, which creates an additional level of complexity in the ILCs family (Locksley, 2009; Chen et al., 2018; Nagasawa et al., 2019; Ricardo-Gonzalez et al., 2020). It turned out that ILC2s protected the body from the effects of invading pathogens, perceiving a signal from epithelial cells passed through effector molecules such as cytokines (GM-CSF, IL-1α, IL-25, IL-33, TSLP), chemokines (CCL17, CCL22), and mediators (ATP, uric acid). Compared to type 1 immune responses to bacterial or viral infections, type 2 immune responses are more complex, coordinating immune reactions to helminths, microscopic particles including pollen, house dust mites, and soluble enzymes such as proteases (Gurram and Zhu, 2019). Activated by the ILC2s effector molecules, they attract dendritic cells, eosinophils, basophils, mast cells, and Th2 lymphocytes (Liu, 2009). The neuropeptide neuromedin U (NMU) can also activate ILC2s through the surface receptor neuromedin U1 (Nmur1); this type of neuroregulation occurs in response to helminth invasion (CardosoChesné et al., 2017; Klose et al., 2017). In addition, ILC2s can be stimulated by estrogen-α (ER-α, Esr1), but estrogen-β (ER-β, Esr2) had no effect on ILC2s-mediated airway inflammation (Cephus et al., 2021). Thus, through the activation of ILC2s, the nervous and endocrine regulation of the type 2 immune response is carried out.

## 4 Conclusion

About 30% of the world’s population suffers from allergic rhinitis, with more than half of the cases associated with allergies to plant pollen. Sensitization to pollen allergens is the cause of allergies not only to plant pollen, but also food allergies, and can also have a negative impact on other diseases, exhausting function of the immune system.

Sensitization to pollen allergens is a complex process and depends on a large number of factors: 1) the state of the immune system of the human body and its genetic predisposition; 2) the composition and properties of allergens; 3) duration of pollen allergen exposition; 4) environmental factors. Disturbed barrier functions of tissues contribute to the transepithelial entrance of allergens and the initiation of allergic inflammation. Plant pollen is a set of molecules of protein, lipid and polysaccharide nature, contains various enzymes and microorganisms that interact with each other and affect the corresponding cell receptors, contributing to inflammation. Some pollen allergens may be pan-allergens present in other sources. As a result, pollen sensitization can lead to food allergies. The study of the intrinsic relationship between the components of pollen and the mechanism of reactivity of immunocompetent cells to recombinant allergens, including a large number of their modifications, makes it possible to identify the checkpoints of the pathological process and develop ways of treatment and allergy prevention.

Involvement of a great number compounds into allergic inflammation made it possible to form the “two-signal hypothesis” of pollen-induced allergic inflammation in which “signal 1,” consisting of innate signaling amplifies “signal 2,” the classical pathway for antigen presentation to T cells, which confer immunological specificity to the immune response. In this case, the removal of signal 1 by prior administration of antioxidants such as ascorbic acid, N-acetylcysteine or tocopherol inhibits allergic airway inflammation. At the same time, cigarette smoke and pollutants can act as triggers of allergic inflammation (Hosoki et al., 2015). With the development of our knowledge about the initiation of a response to allergenic molecules, the diversity of the involved populations of immunocompetent cells, signals and mediators became obvious. The dependence of the activation type on the structure of the allergen, microenvironment, and duration of exposure was also revealed. At present, structures of several thousand allergens have been determined, and the mechanism of action has been investigated for many of them (Bogdanov et al., 20212058; Finkina et al., 2021; Melnikova et al., 2021). In order to take into account the risk factors for an occurrence of allergic diseases, databases of allergens are created and improved (Guryanova, 2018; van Ree et al., 2021). Main characteristics of allergens including molecular masses, epitope structures, cross-reactivity, presence in foods and geographical distribution are collected there. Surprisingly, the purified recombinant proteins of birch pollen were not able to induce an immune response, while the pollen extract caused it (Aglas et al., 2018). The recombinant Phl p 5 also did not induce IL4-producing Th2 cells, compared with *in vivo* test of complete Timothy pollen extract (Araujo et al., 2020). Thus, and subsequent allergic inflammation is due to the composition of the pollen, and not due to the inherent allergenicity of the proteins.

The most important concluding remarks

• Previously, it has been shown that the pollen grain has a complex composition in which allergenic proteins are embedded in a heterogeneous matrix with many bioactive molecules acting simultaneously during allergic sensitization process.
• The main allergens from tree, grass and weed pollen are proteins belonging to the classes of the Bet v 1 homologs, LTPs, profilins, polcalcins, β-expansins and to the so called group 5 allergens.
• Due to the ubiquitous presence not only in pollen, but also in plant foods, some of these proteins having a high structural homology and epitope similarity are panallergens causing pollen-pollen and pollen-food cross-allergic reactions.
• Currently, it is clear that not only structural and physicochemical features and inherent immune-modulating properties of protein allergens determine their allergenic potency.
• Such allergens as the Bet v 1 homologs and LTPs are able to bind hydrophobic ligands that may affect manifestation of their allergenic properties.
• At the same time, pollen microbial and plant lipids due to their adjuvant properties may play an important role in the sensitization to both lipid-binding and non-lipid-binding allergens.
• Now, it is supposed that pollen sensitization resulted from complex interactions between the innate immune cells, allergens and pollen-derived adjuvants of different nature co-delivered with them.
• The question remains open whether lipids have own immunological properties synergizing allergic inflammation.
• Signal transduction cascades induced by allergens and their natural ligands should be investigated further.
• Epithelial cells of respiratory and intestinal tracts are major players in allergic inflammation leading to allergic sensitization through direct stimulation of antigen-presenting cells and indirect activation of innate lymphoid cells (ILC2).
• The first step in the sensitization process goes along with an activation of PRR, biosynthesis of pro-inflammatory cytokines (IL-1, IL-6, IL-8 and TNF-α) and pro-allergic alarmins (TSLP, IL-33 and IL-25).
• It was found that primary and tertiary structures of allergens affected processing, presentation on MHC class II molecules, loaded on various HLA phenotypes and, as a result, activated various immune cells (DC, M2 macrophages, Th2, Teff, Treg).
• Pollen activation of the Th2 immune response and IgE production in pollen-allergic individuals is of much interest, while the Th1 response is predominant in healthy individuals.
• Now, it is supposed that in addition to the Th2-mediated, another way of activating of the immune response without the participation of lymphocytes is also possible as ILC2 cells, activated via IL-33, cause eosinophilic inflammation.
• Taken together, structures of allergens, interaction with their natural ligands and accompanying molecules, complexity of allergen exposure conditions, the presence of environmental cofactors play a symphonic role in allergic pollen sensitization.
• Fundamental studies of the sensitization mechanisms provide the basis development of drugs for treatment and prevention of allergic inflammation.
